# Supplementary material for: Dynamic transcriptomic profiles of zebrafish gills in response to zinc supplementation
Source: BMC Genomics. 2010 Oct 11;11:553. doi: 10.1186/1471-2164-11-553 (PMC3091702; doi:10.1186/1471-2164-11-553)
Supplement: Additional file 2 — Interactive Direct Interaction Network representing the molecular interactions between zinc, copper, iron, calcium and proteins encoded by transcripts changed by zinc supplementation. Mini web-site containing index.html and hyperlinked pages in subdirectory describing a Direct Interaction Network automatically generated based on curated interactions contained within the proprietary PathwayArchitect database. Ovals represent proteins and the circles symbolize metal ions. Objects are coloured by their abundance in zebrafish at the time-point they were significantly different from the control is a scale from -4 fold (dark green) to +4 fold (dark red). Where significant differences were found at more than one time-point, the colour overlay shows expression at the first instance. Dark blue squares denote 'binding', and light blue squares 'expression'; green squares stand for 'regulation', green diamonds for 'metabolism', and green circles for 'promoter binding'. Arrow heads indicate directionality of the interaction where annotated. All nodes and edges can be further interrogated by selecting the relative area of the image. [file 1471-2164-11-553-S2.zip › PathwayArchitect Zn xs DIN/74828.html]

# PROTEIN: DDX10

|  |  |
| --- | --- |
| Name | DDX10 |
| Type | PROTEIN |
| Description | DEAD (Asp-Glu-Ala-Asp) box polypeptide 10 |
| Note | DEAD box proteins, characterized by the conserved motif Asp-Glu-Ala-Asp (DEAD), are putative RNA helicases. They are implicated in a number of cellular processes involving alteration of RNA secondary structure such as translation initiation, nuclear and mitochondrial splicing, and ribosome and spliceosome assembly. Based on their distribution patterns, some members of this family are believed to be involved in embryogenesis, spermatogenesis, and cellular growth and division. This gene encodes a DEAD box protein, and it may be involved in ribosome assembly. Fusion of this gene and the nucleoporin gene, NUP98, by inversion 11 (p15q22) chromosome translocation is found in the patients with de novo or therapy-related myeloid malignancies. |
| Alias | HRH-J8 |
|  | DDX10 |
|  | AI646054 |
|  | DEAD box-10 |
|  | MGC91294 |
|  | Ddx10 |
|  | 4632415A01Rik |
|  | DEAD/H (Asp-Glu-Ala-Asp/His) box polypeptide 10 (RNA helicase) |
|  | DEAD-box protein 10 |


---

|  |  |
| --- | --- |
| GO ID | GO:0003723 |
|  | GO:0005524 |
|  | GO:0003724 |
|  | GO:0004386 |
|  | GO:0003676 |
|  | GO:0008026 |
|  | GO:0016787 |
|  | GO:0000166 |


---

|  |  |
| --- | --- |
| MIM | MIM:601235 |


---

|  |  |
| --- | --- |
| Connectivity | 18 |


---

|  |  |
| --- | --- |
| Entrez ID | 77591 |
|  | 1662 |


---

|  |  |
| --- | --- |
| Agilent ID | A\_14\_P104826 |
|  | A\_32\_P93149 |
|  | A\_14\_P120927 |
|  | A\_53\_P127272 |
|  | A\_23\_P32938 |
|  | A\_53\_P135708 |
|  | A\_51\_P384609 |
|  | A\_52\_P335968 |
|  | A\_53\_P145625 |
|  | A\_53\_P108816 |
|  | A\_14\_P103603 |
|  | A\_14\_P116089 |
|  | A\_53\_P132131 |
|  | A\_14\_P135026 |


---

|  |  |
| --- | --- |
| Pathway | Zn xs inventory |
|  | Zn xs DIN |


---

|  |  |
| --- | --- |
| UniGene | Hs.525115 |
|  | Mm.133560 |


---

|  |  |
| --- | --- |
| Affymetrix Probeset ID | 115675\_at |
|  | 1428563\_at |
|  | 1443061\_at |
|  | 1563522\_at |
|  | 1452838\_at |
|  | 163619\_at |
|  | 169102\_r\_at |
|  | 169612\_r\_at |
|  | 204977\_at |
|  | 138859\_f\_at |
|  | 831\_at |
|  | 99878\_at |
|  | g13514830\_3p\_at |
|  | Msa.5506.0\_s\_at |
|  | 117237\_at |
|  | U28042\_at |
|  | AFFX-hum\_alu\_at |
|  | hum\_alu\_at |
|  | 74247\_at |
|  | Hs2.376837.1.S1\_3p\_at |
|  | TC32449\_at |


---

|  |  |
| --- | --- |
| EC Number | EC 3.6.1.- |


---

|  |  |
| --- | --- |
| GO Function | hydrolase activity |
|  | RNA helicase activity |
|  | RNA binding |
|  | helicase activity |
|  | nucleotide binding |
|  | nucleic acid binding |
|  | ATP-dependent helicase activity |
|  | ATP binding |


---

|  |  |
| --- | --- |
| Nucleotide | BC049217 |
|  | AK019495 |
|  | U28042 |
|  | AB040537 |
|  | BC049261 |
|  | AI646054 |
|  | NM\_004398 |
|  | AW121262 |
|  | BC093654 |
|  | AK166787 |
|  | BC055481 |
|  | BC080729 |
|  | BC023303 |
|  | AK045032 |
|  | AB209884 |
|  | XM\_284494 |
|  | AK052540 |
|  | BC064921 |
|  | BC093656 |
|  | BC091521 |
|  | AW124460 |


---

|  |  |
| --- | --- |
| Protein | AAH49261 |
|  | AAH93656 |
|  | AAH93654 |
|  | AAH23303 |
|  | AAH49217 |
|  | AAH91521 |
|  | BAB18536 |
|  | BAE39018 |
|  | BAC32191 |
|  | NP\_004389 |
|  | AAH55481 |
|  | BAC35031 |
|  | XP\_284494 |
|  | AAH80729 |
|  | Q13206 |
|  | AAC50823 |
|  | Q80Y44 |
|  | BAD93121 |


---

|  |  |
| --- | --- |
| Organism | Mammal |


---

|  |  |
| --- | --- |
| Location | chromosome 9, 9 A5.3 (Mus musculus) |
|  | chromosome 11, 11q22-q23 (Homo sapiens) |


---

|  |  |
| --- | --- |
